# Is the Brain's Inertia for Motor Movements Different for Acceleration and Deceleration?

**DOI:** 10.1371/journal.pone.0078055

**Published:** 2013-10-21

**Authors:** Bhim M. Adhikari, Kristen M. Quinn, Mukesh Dhamala

**Affiliations:** Department of Physics and Astronomy, Neuroscience Institute, Center for Behavioral Neuroscience, Georgia State University, Atlanta, Georgia, United States of America; Universiteit Gent, Belgium

## Abstract

The brain's ability to synchronize movements with external cues is used daily, yet neuroscience is far from a full understanding of the brain mechanisms that facilitate and set behavioral limits on these sequential performances. This functional magnetic resonance imaging (fMRI) study was designed to help understand the neural basis of behavioral performance differences on a synchronizing movement task during increasing (acceleration) and decreasing (deceleration) metronome rates. In the MRI scanner, subjects were instructed to tap their right index finger on a response box in synchrony to visual cues presented on a display screen. The tapping rate varied either continuously or in discrete steps ranging from 0.5 Hz to 3 Hz. Subjects were able to synchronize better during continuously accelerating rhythms than in continuously or discretely decelerating rhythms. The fMRI data revealed that the precuneus was activated more during continuous deceleration than during acceleration with the hysteresis effect significant at rhythm rates above 1 Hz. From the behavioral data, two performance measures, tapping rate and synchrony index, were derived to further analyze the relative brain activity during acceleration and deceleration of rhythms. Tapping rate was associated with a greater brain activity during deceleration in the cerebellum, superior temporal gyrus and parahippocampal gyrus. Synchrony index was associated with a greater activity during the continuous acceleration phase than during the continuous deceleration or discrete acceleration phases in a distributed network of regions including the prefrontal cortex and precuneus. These results indicate that the brain's inertia for movement is different for acceleration and deceleration, which may have implications in understanding the origin of our perceptual and behavioral limits.

## Introduction

Rhythm is an essential part of our daily lives as almost all common activities require an element of timing. We can create, maintain and change an incredible number of slow, fast, simple or intricate movement rhythms. For example, tapping your feet, clapping along, dancing or playing an instrument all are rhythmic activities intimately coordinated with a timed external cue, music. The coordination of rhythmic movement with an external rhythm is called sensorimotor synchronization (SMS) [1]. Previous neuroimaging studies demonstrate that SMS involves a distributed network of brain regions with responsibilities ranging from the integration of sensory stimuli to motor planning and execution [2]–[4]. This study aims to shed light on how the brain achieves its precise timing, is it more difficult to achieve SMS with accelerating or decelerating rhythms, and what neuronal features set the behavioral limits on the speed and accuracy of SMS. Answers to these questions will have implications not only for understanding the origin of the brain's cognitive ‘inertia’, but also for rehabilitation efforts in movement disorders including Parkinson's disease, Huntington's disease and traumatic brain injury.

SMS paradigms are widely used because of their simplicity, convenience, and relevance. Previous studies on rhythm have explored neural representations of integer and non-integer ratio rhythms [5], neural correlates of the motor rhythm complexity [4], [6], [7], neural correlates of rhythmic versus discrete movements [7], the neural basis of human dance [8], and brain networks for integrative rhythm formation [4]. The basal ganglia, cerebellum, and various parts of the cortex have been shown to be involved in perceiving and generating simple to complex movement rhythms. The basal ganglia have been related to basic timing and sequencing aspects of rhythmic motor movements [4], [9], whereas the cerebellum and the cortical sensorimotor areas have been related to temporal complexity or the fine-tuning of rhythms [6] and the sensorimotor integration for optimizing movements [4], [9], [10]. It is clear that the cortico-basal ganglia and cortico-cerebellar circuits are involved not only in rhythmic movement generation, but also in various aspects of rhythmic perception and learning [11]. Damage to these circuits impairs timing abilities [12]–[15], further supporting their crucial role in rhythm perception and production. These impairments are associated with a wide variety of neurological disorders such as Parkinson's disease, schizophrenia, attention deficit hyperactivity disorder (ADHD) and autism [16]–[18]. Therefore, we expect to see these brain regions recruited during the SMS task. Previous studies have found precuneus activity to be modulated by greater complexity, difficulty or load of task [19], [20]. Thus we hypothesize additional activations, particularly precuneus activity, will be associated with the more difficult phases of the SMS task, i.e. decelerating rather than accelerating phases.

Movement rate has been linked to nonlinear brain BOLD (blood-oxygen level dependent) responses [21], [22]. Berns and colleagues have reported a nonlinear effect - hysteresis or a history-dependent effect - in brain BOLD responses associated with linearly changing movement rates [23]. Fraisse and Voillaume [24] found that subjects finger-tapping in a pseudo-SMS task (one in which their tapping rate actually generated the auditory metronome) accelerated their tapping progressively, falsely believing that the metronome's rate was increasing. Even those participants who were made aware that their tapping rate dictated the metronome's rate substantially accelerated their tapping speed [24]. In light of these findings, we hypothesized that SMS with an accelerating rate would be easier than with a decelerating rate. Other exploratory questions include: is the brain's inertia for motor movements different for accelerating and decelerating rates? Are these history effects related to behavioral responses? Are there differences in the brain activity of the basal ganglia and the cerebellum between decelerating and accelerating rates of rhythms? Is there a difference in the brain's response for continuously changing versus discretely changing rates of rhythms? This work addresses these questions.

The different levels of brain processes involved in initiation, execution, maintenance and termination of a learned behavior [25], [26] suggest that the central nervous system has an inherent resistance to changes in brain states. This intrinsic resistive property can be thought of as the brain's inertia and is hypothesized to originate from the nonlinear neuronal characteristics of cerebral activity. Neural activity at the level of the single cell is endowed with history-dependent effects [27]–[29] and, on a larger scale, the activity of brain networks underlying perception and behavior is influenced by history or prior context [30], [31]. SMS to an external metronome is a simple but efficacious task paradigm to evaluate the impulse-response relationship and history or context-dependent basis of the hysteresis effects. Using visual metronome and SMS paradigm, we can assess whether the brain's inertia changes for different rates of rhythms, and whether there is a difference in motor coordination with an increasing or with a decreasing rhythm rate. In physics, Newton's first law introduces the notion of objects' inertia as the tendency to resist changes in their state of motion. Newton's second law provides a quantitative link of mass (measure of inertia) with force and acceleration (or deceleration). Dynamical systems theory predicts the existence of hysteresis in responses of bi-stable (or multi-stable) dynamical systems, like the neural systems in the brain, for increasing and decreasing impulses [32]. In this study, these physics concepts were employed to compare the brain's inertia for accelerating and decelerating rhythms by matching the physical parameters of the sensory input and manipulating only the history of stimulation and perception.

Coordinating movements are often categorized as discrete or continuous sequences. Discrete movements, such as reaching out and grasping an object, are believed to require different timing control mechanisms than continuous movements, such as rhythmic finger flexion and extension [7], [33]. In this experiment, the movement rhythms were manipulated in two trials: continuous sinusoidal variation and discrete stair-like variation, which attempted to follow the similar classification scheme of motor movements. Using these concepts, we hypothesized that discrete SMS would recruit different brain regions than continuous SMS.

We recorded timing sequences of performed rhythms and fMRI brain signals while subjects participated in the SMS task, matching their finger tapping to a visual metronome whose rate followed a sinusoidal curve either continuously or discretely. Thus there were both discrete and continuous, acceleration and deceleration phases of the task. The behavioral performance and fMRI data allowed us to isolate and compare the brain's inertia during accelerating and decelerating rates of rhythms during continuous and discrete cases of the SMS task. The tasks either visuospatial information processing or memory-related cognitive activities [19] or motor-coordination [20] involve precuneus activity. The precuneus has widespread anatomical connection to higher association cortical and subcortical areas [19]. So, we selected the observed activated brain region, the precuneus, in our visual stimulus-response sequence SMS task to explore more.

## Methods

### Subjects

Thirteen right-handed subjects, with no history of neurological disease or musical expertise, participated in this study. Due to poor performance (as measured by average synchrony index <0.15) or head movement (>2 mm), five were excluded leaving eight subjects included in analysis. All subjects were right handed and between the ages of 23 and 37 years old. Signed, informed consent was collected from each subject prior to participating in the study. Institutional Review Board (IRB) of Emory University approved the experimental protocol and Georgia State University IRB approved the reanalysis of the data.

### Task Paradigm

**Figure 1:**
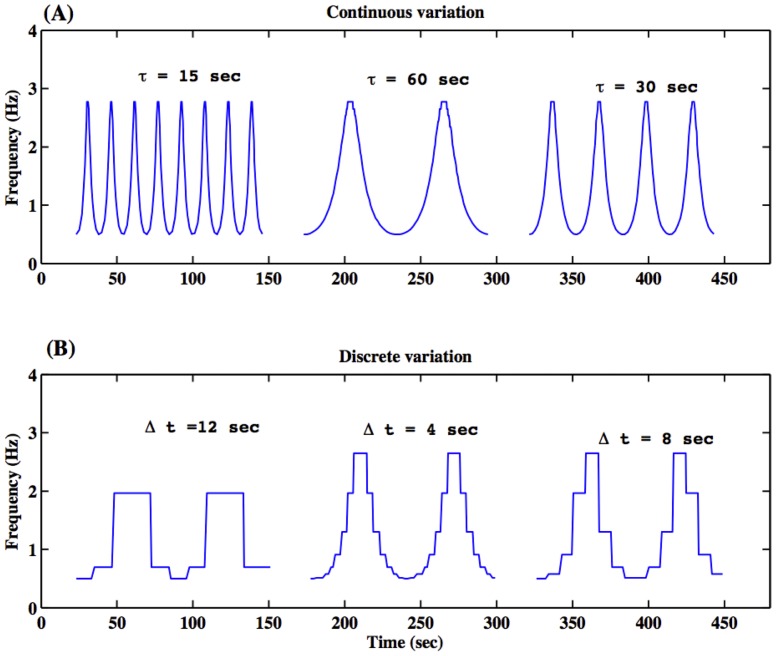
Task paradigm: frequency of tapping versus time. Smooth sinusoidal variation of rates of tapping is shown in the upper trace and discrete stair-like variation of rates of tapping is shown in the lower trace. There were three cycles of tasks separated by no task (rest) periods. The order of these three task cycles was randomized for different subjects. Sinusoidal variation had three cycles 15 sec, 60 sec and 30 sec (top panel), and discrete variation had three time-widths 4 sec, 8 sec, 12 sec embedded in a 60 sec-cycle of continuous variation (bottom panel).

The experiment consisted of two tasks: rhythmic finger tapping following a continuous sinusoidal increase and decrease of rate (Fig. 1, upper trace), and rhythmic finger tapping following a sinusoidal stair-like (discrete) increase and decrease of rate (Fig. 1, lower trace). The first task will be referred to as the continuous case and the second as the discrete case. The order of these cases was randomized across subjects. A synchronization accuracy score assessed the behavioral performance on each SMS task, measuring how well the subject could synchronize their timing of tapping with the metronomes'. The two cases were performed in two functional runs. Each run was 470 seconds long (7 min 50 sec). In the fMRI scanner, subjects used their right index finger for tapping on a response box in synchrony to visual cues. The interval was continuously time-modulated based on the sine function. Subjects were instructed to attempt to tap in synchrony with a visual metronome; they tapped the button on the response–box in synchrony with a small blue square's appearance on the black display background. The subject followed the sinusoidal rhythm faster and slower with accelerating and decelerating rate governed by the flashed visual cues. Each of the two cases (continuous and discrete) had three different cycles. The continuous case had cycles of 15 sec, 30 sec, and 60 sec, during which the frequency changed approximately from 0.5 Hz to 3 Hz (as shown in Fig. 1). Some find the lower bound of 0.5 Hz slightly outside the synchronization range [34]; however, this range of tempi was chosen as previous studies have shown synchronization as similarly low rates of 0.6 Hz [35], [36]. The discrete case had three time-widths 4 sec, 8 sec, 12 sec embedded in a 60 sec-cycle of continuous variation (Fig. 1). These modulation rates were established so that subjects would have to adjust their tapping sequences. The cycle presentation order was randomly sequenced for each subject. Each case began and ended with 22 sec of rest and had 24 sec of rest between each of the three finger tapping cycles. Response times (times when the subject pressed the button) were recorded throughout.

### Image acquisition

The imaging was done on a 1.5 T Philips Intera scanner. After acquiring a high-resolution T1-weighted anatomical image, two whole-brain functional runs were performed with 235 scans in each run with the following parameters: echo-planar imaging, gradient recalled echo sequence; TR = 2000 ms; TE = 40 ms; flip angle = 90°; 64 64 matrix, 24 axial slices each of 5 mm thickness acquired parallel to the anterior-posterior commissural line for the measurement of the T2*-weighted blood oxygenation level-dependent (BOLD) effect [37], [38].

### Behavioral data analysis

Using the recorded response times and time-modulated visual cues, we assessed the rate at which subjects tapped their fingers and how well they were able to synchronize their finger tapping with the external metronome. The blue box flashing on the display screen acted as a visual metronome, indicating the correct time for the subject to tap on the response box. Two measures were used for analysis, the rate of tapping and the synchrony index (S_i_). The synchrony index measures how well participants synchronized their taps with the cues and is defined as follows: 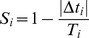, where t_i_ is the time interval between the visual cue and the performed finger-tap, and T_i_ is the time-interval between consecutive visual cues in the i^th^ interval. We computed separate synchrony index measures for acceleration and deceleration phases of the rhythms from both continuous and discrete cases. Wilcoxon rank-sum tests were used to determine significant differences in behavioral performance between the acceleration and deceleration phases of the tasks. A lower synchrony index value indicates a lower accuracy on the task, and therefore an inferred higher level of difficulty. After completing the experiment, all subjects reported greater difficulty in SMS with the deceleration portions of the task than the acceleration portions for continuous variation. This synchrony measure and subjects' self-reports allowed us to compare the difficulty levels in following an accelerating or decelerating rhythm. The common systematic error called negative asynchrony was observed. This effect commonly occurs when the subject's tap response precedes the stimulus metronome [39]. The more negative the mean asynchrony score the more time elapsed between the anticipatory tap and the stimulus. We calculated the mean of the negative asynchrony score normalized by the time periods between visual cues from the subjects' performance in each of the four conditions, discrete accelerating, continuous accelerating, discrete decelerating and continuous decelerating.

### Brain data analysis

fMRI images were preprocessed and analyzed using Statistical Parametric Mapping (SPM8) (Wellcome Trust, London; [40], [41], http://www.fil.ion.ucl.ac.uk/spm). Motion correction was performed using a six-parameter rigid-body transformation; all of the eight subjects included in analysis had less than 2 mm translation in all directions and less than 1.0° of rotation about the three axes. The mean of the motion-corrected images was co-registered to the individual's 24-slice structural image using a 12-parameter affine transformation. The images were spatially normalized to the Montreal Neurological Institute (MNI) template [42] by applying a 12-parameter affine transformation, then underwent a nonlinear warping using basis functions [43]. Images were then smoothed with an 8-mm isotropic Gaussian kernel and high-pass-filtered in the temporal domain to remove a low-frequency trend.

A random effects, model-based, statistical analysis was performed with SPM8 in a two-level procedure. At the first level, two separate general linear models (GLM) of the form: 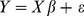, were specified for each participant, where ***X = [1, X_1_, X_2_, …]***, 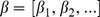 and 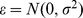. In the first GLM model, ***X = [1, X_1_, X_2_, …]*** was a design matrix that included task-rest conditions and time-courses of 6 motion parameters, total of 8 regressors. In the second model, the ***X***-matrix included rates of tapping, instantaneous synchrony indices during acceleration and deceleration phases, and time-courses of 6 motion parameters, making total regressors equal to 10, separately for continuous and discrete cases. 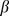
's are the corresponding estimated coefficients for the columns of X, and 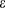
represents the unexplained variance term. The following contrasts were evaluated: task versus rest, rate of tapping in acceleration versus rate of tapping in deceleration or vice-versa, and synchrony in acceleration versus deceleration and vice-versa in both continuous and discrete cases. These contrasts, such as rate of tapping in deceleration versus rate of tapping in acceleration, were designed to highlight brain regions whose activity increases during the first condition (e.g. deceleration) more than during the second condition (acceleration). These individual contrast images (a total of 10 contrasts, including tap-versus-rest contrast) were then entered into a second-level analysis, using a separate one-sample *t* test. The resulting summary statistical maps were subjected to an initial cluster forming threshold p<0.001 (uncorrected) and a cluster size k >10 voxels. These maps were overlaid on a high-resolution structural image in MNI orientation for displaying fMRI activations. For the analysis of hysteresis effects, time courses were extracted from a spherical region of 6 mm radius centered at the peak activity voxel using MarsBaR [44].

## Results

### Behavioral response

**Figure 2:**
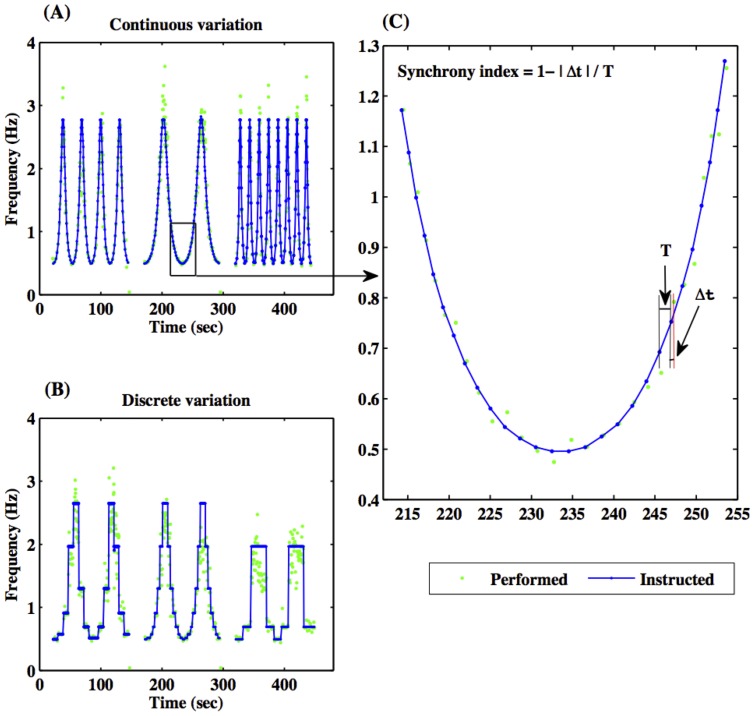
Tapping rates in Hz versus time in seconds (blue for metronomes, green for performed taps). (A) Continuous sinusoidal variation of rhythm rates: instructed with visual cues (blue) and performed taps (green). (B) Discrete sinusoidal variation of rhythm rates with time: instructed with visual cues (blue) and performed taps (green). (C) A blow-up of a portion from plot (A) showing how the synchrony measure was defined.

Metronome rates and finger-tapping performance were calculated from the onset of the visual cues presented on the screen and the times of the subjects' finger-tapping button responses. Fig. 2 (A-C) shows representative plots from a subject for the presented visual cues (blue dots) and the performed behavior (green dots). Fig 2 (A) depicts the continuous sinusoidal and (B) the discrete frequency variation cases, and (C) is a blow-up of (A) to illustrate the definition of the synchrony measure. The instantaneous synchrony measure, referred to as the synchrony index is defined as: 
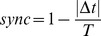
, where 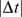 is the time interval between a cue and a response, **T** is the time interval between visual cues (the maximum time allotted for the subject to react to that specific cue), and 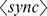 is an average over many repeated responses. Sync can range from 0 to 1, where 0 means no synchrony and 1 means a perfect synchrony between a visual cue and subject's response.

**Figure 3:**
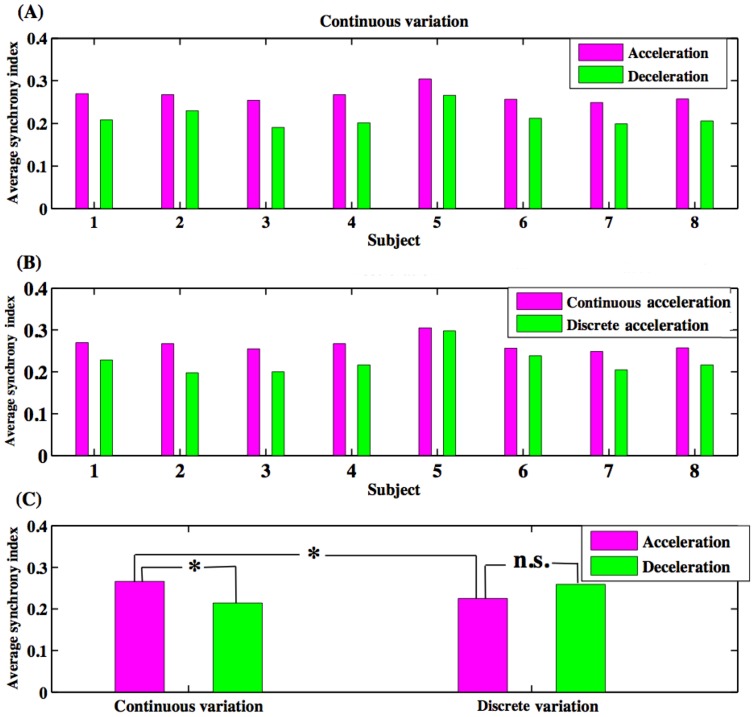
Average synchrony between metronomes and behavioral responses during acceleration and deceleration phases of the tasks. (A) Individual subject–level average synchrony index for the continuous case, (B) individual subject-level average synchrony index for acceleration phase in the continuous and discrete cases, and (C) group-level averages of synchrony indices and significant levels (* means p<0.01, n. s. means not significant).

From Fig. 2 (A–C), it is clear that subjects had higher synchrony indices (indicated in green) following visual cues (indicated with blue dots) at lower frequencies than at higher frequencies. By using the average synchrony index (sync) as defined above, we found that there was a significantly higher synchrony index for increasing rates of rhythms (acceleration) than for decreasing rhythms (deceleration) in continuous sinusoidal variation for all subjects [Fig. 3 (A) and 3 (C)]. Average synchrony indices between accelerating and decelerating phases were not significantly different however in the discrete case (Fig. 3 (C)). The synchrony index during acceleration in the continuous case was significantly higher than the synchrony index during acceleration in the discrete case [Fig. 3 (B–C)]. These behavioral results are consistent with what subjects reported in their post-task response: that deceleration was more difficult than acceleration in the continuous case and that the discrete case was more difficult than the continuous case. The Wilcoxon rank-sum tests showed that (i) the difference of sync between deceleration and acceleration was significant at p<0.01 and (ii) the difference of sync between continuous and discrete cases during acceleration was significant at p<0.01 [Fig. 3 (C)].

Means of the negative asynchrony scores were greater in magnitude for the decelerating cases. Discrete deceleration had a significantly lower negative asynchrony score than the discrete acceleration case (p<0.01) and continuous deceleration had a significantly lower negative asynchrony score than the continuous acceleration case (p<0.01). There were no significant differences in negative asynchrony between accelerating cases of discrete versus continuous rhythms, or decelerating cases of discrete versus continuous. These results make intuitive sense and support earlier conclusions made from average synchrony index scores that suggest synchronization with an external metronome is easier with accelerating rates and more difficult with decelerating rates. The negative asynchrony scores strengthen our hypothesis that though completing the same finger-tapping task, the brain shows a hysteresis effect; participants' SMS ability was affected by the historical rate of stimuli. Subjects have enhanced synchronization performance during accelerating versus decelerating rhythm rates.

### Brain response

We evaluated various contrasts: (i) task (acceleration and deceleration) versus rest, (ii) acceleration versus deceleration and vice versa, (iii) frequency of finger-tapping in acceleration phase versus frequency of finger-tapping in deceleration phase and vice-versa, and (iv) sync in acceleration versus deceleration and vice-versa in both continuous and discrete cases. Table 1 lists the brain activations associated with the task versus rest contrast and those that were associated with the significant behavioral results. These activations were subjected to an initial cluster forming threshold p<0.001(uncorrected) and a cluster size k >10, and were corrected for multiple comparisons using the AlphaSim command in AFNI ([45]; B. D. Ward, http://afni.nih.gov/afni/docpdf/AlphaSim.pdf). All the activations survive the significance of corrected p<0.05 (multiple comparisons) with the individual voxel threshold probability threshold of 0.05.

**Table 1:** Significant brain activations for various contrasts.

Contrast	Brain region	Cluster size	Voxel t (z-equivalent)	MNI coordinates
Task versus Rest	L PM **	20	6.94 (3.69)	−48, −7, 52
(continuous and discrete cases combined)	R SN ***	17	6.80 (3.66)	3, −16, −20
Deceleration versus acceleration (continuous variation)	L Precuneus ***	11	5.64 (3.36)	−3, −58, 28
Rate in deceleration versus rate in acceleration	R cerebellar vermis***	59	9.30 (4.14)	30, −55, −23
(continuous variation)	L cerebellar vermis***	32	6.90 (3.68)	−24, −64, −23
	L STG*	18	7.74 (3.63)	−33, −1, −20
	R PHCG *	13	6.32 (3.54)	27, −25, −14
Synchrony in acceleration versus synchrony in	L MeFG ***	31	15.85 (4.90)	−9, 53, −2 —
deceleration (continuous variation)	R PHCG **	14	8.07 (3.93)	27, −13, −29
	L uncus **	15	7.84 (3.88)	27, −1, −35 24,
	R OLG **	14	7.53 (3.82)	76, −2
Synchrony in continuous case versus synchrony in	R MFG ***	20	10.11 (4.27)	21, −7, 58
discrete case (acceleration phase)	L MeFG *	10	6.40 (3.56)	−15, 2, 52
	R precuneus ***	17	6.21 (3.51)	15, −70, 34
	R PCC *	11	5.61 (3.35)	12, −58, 13

The t-map of each contrast was corrected for multiple comparisons using the AlphaSim command in AFNI (Cox, 1996); B. D. Ward, http://afni.nih.gov/afni/docpdf/AlphaSim.pdf). The individual voxel threshold probability threshold was set to be 0.05. Abbreviations: L =  left, R =  right, PM =  the primary motor cortex in the hand area, SN =  substantia nigra, PCC =  posterior cingulate cortex, STG =  superior temporal gyrus, PHCG =  parahippocampal gyrus, MeFG =  medial frontal gyrus, OLG =  occipital lingual gyrus, and MFG =  middle frontal gyrus. *: p<0.05; **: p<0.01; ***: p<0.001 (AlphaSim correction).

**Figure 4:**
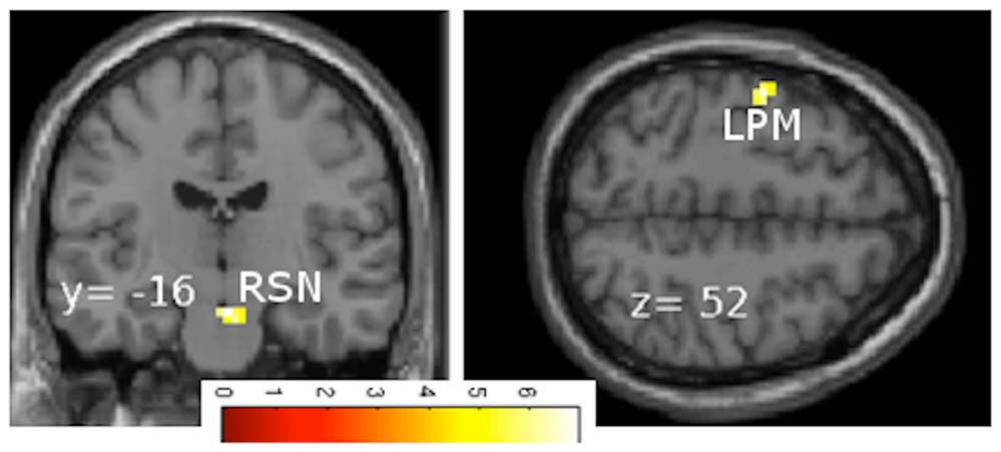
Task > rest contrast in continuous and discrete variations. The left primary motor cortex (LPM) and right substantia nigra (RSN) are active for synchronizing motor movements with variable rhythm rates.

**Figure 5:**
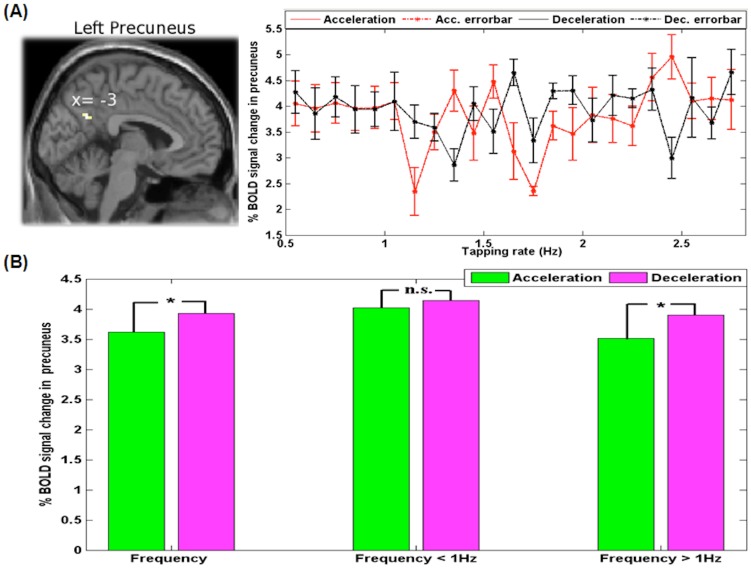
Deceleration > acceleration in continuous rhythm rate variation. (A) The left precuneus activity and group mean time courses with error bars (standard error of the mean) extracted from precuneus during acceleration and deceleration, (B) mean % BOLD signal changes during acceleration and deceleration.

**Figure 6:**
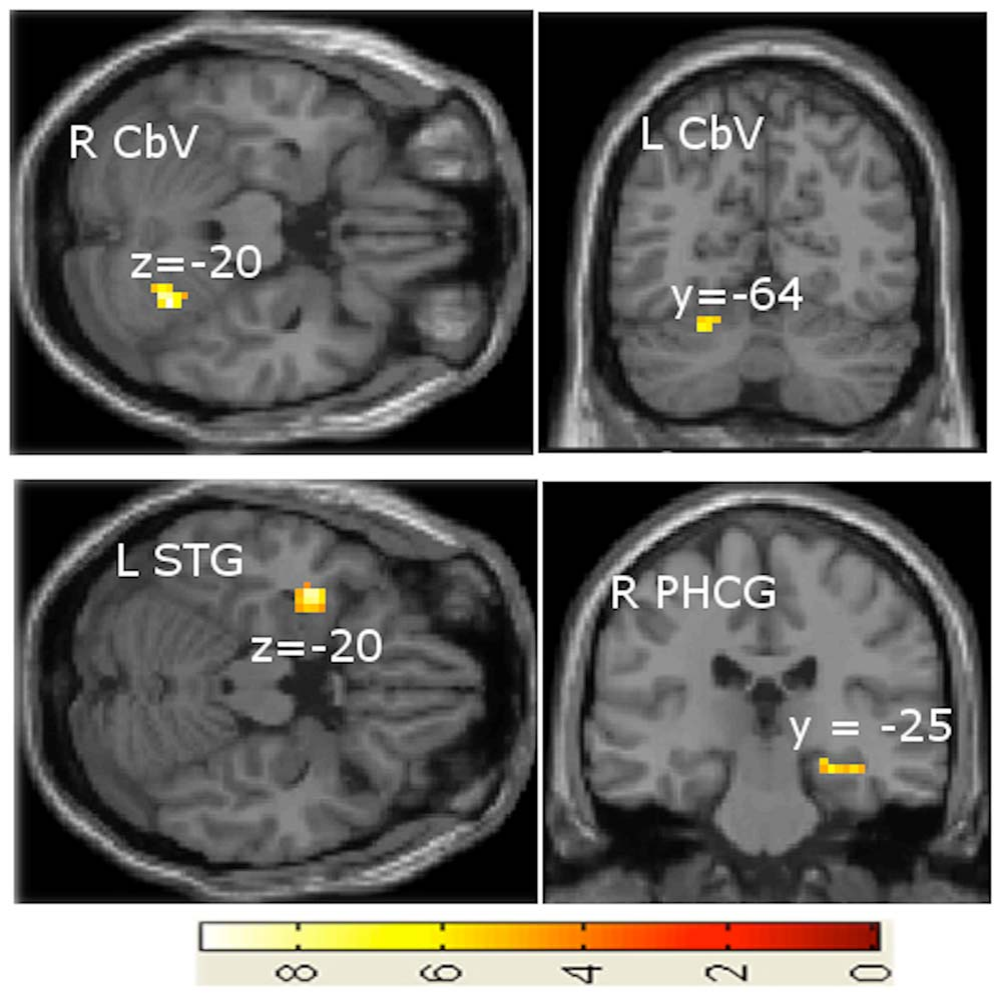
Brain activations associated with decelerating rate > accelerating rate contrast in continuous variation. Abbreviations: L = left, R = right, CbV = cerebellar vermis, STG = superior temporal gyrus, PHCG = parahippocampal gyrus.

**Figure 7:**
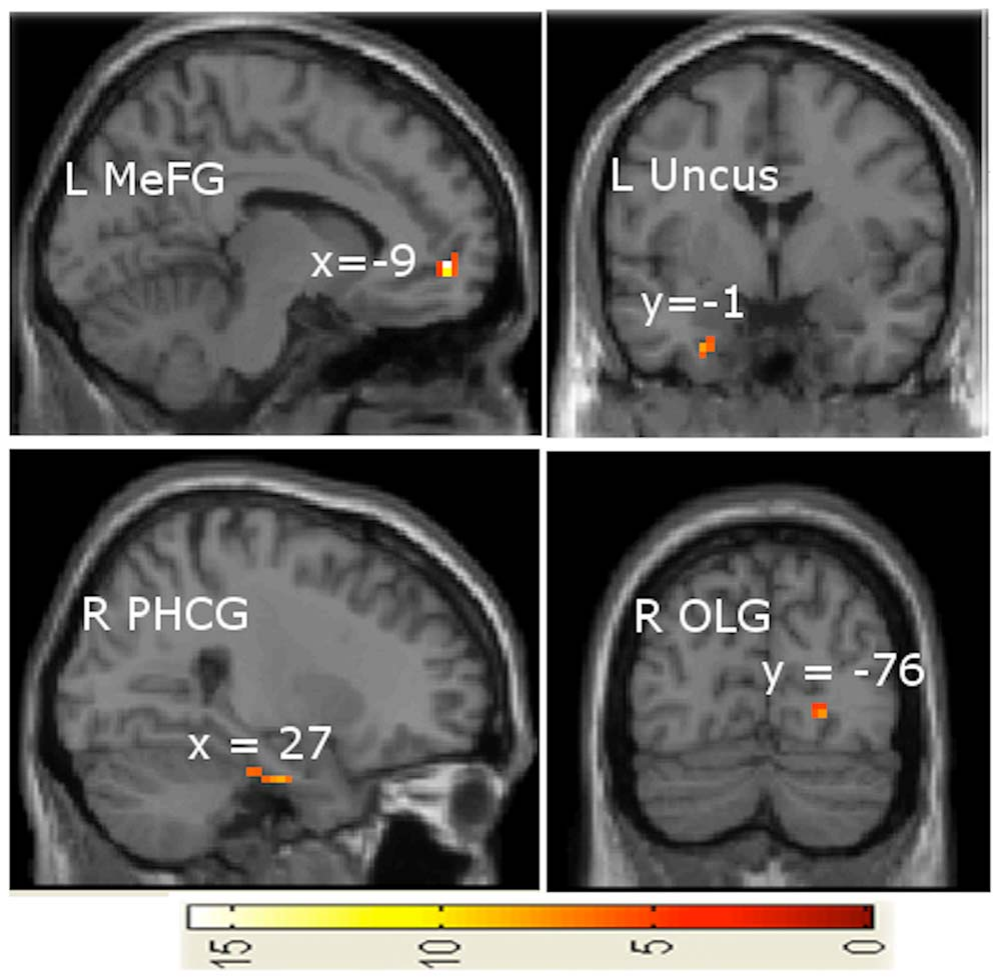
Brain activations for synchrony in acceleration > synchrony in deceleration during the continuous variation. Abbreviations: L =  left, R =  right, MeFG =  medial frontal gurus, PHCG =  parahippocampal gyrus, OLG =  occipital lingual gyrus.

**Figure 8:**
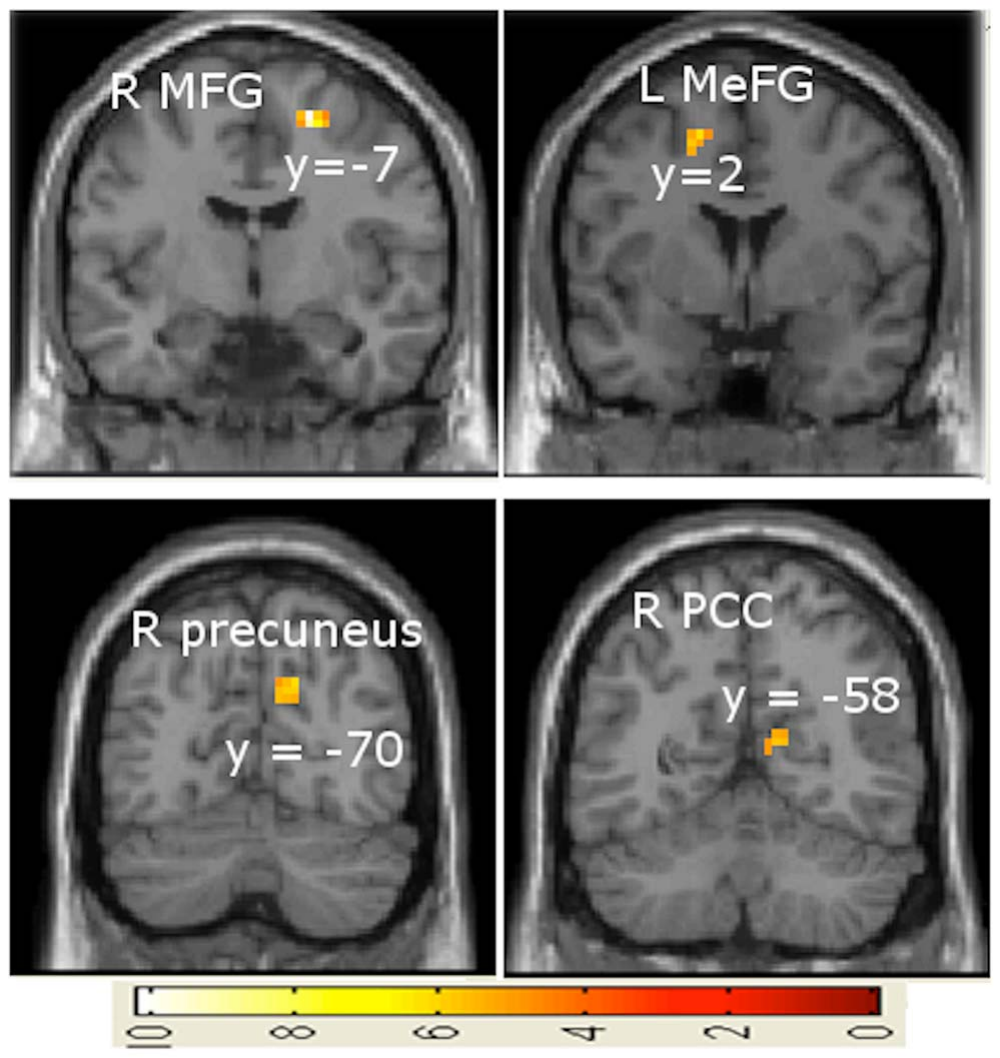
Brain activations for synchrony in continuous variation > synchrony in discrete variation during acceleration phase. Abbreviations: L =  left, R =  right, MFG =  middle frontal gurus, MeFG =  medial frontal gyrus, PCC =  posterior cingulate cortex.

The SMS task versus rest contrast applied to both continuous and discrete cases revealed significant brain activations in the left primary motor cortex (precentral gyrus) in the hand area and in the right substantia nigra (Fig. 4). The deceleration versus acceleration contrast in the continuous case showed left precuneus activity (Fig. 5, first panel). Though the selection of region-of-interest (ROI) was based on the activation clusters, this precuneus ROI survived the multiple comparisons correction across the voxels of the whole brain at significance p<0.05 based on AlphaSim in AFNI. In addition, this ROI had 34 voxels and the adjusted p with Bonferroni correction came out to be p = 0.017 (p<0.05). The ROI analysis shows that the BOLD response is significantly different for deceleration and acceleration, especially for rates above 1 Hz (Fig. 5, first and second panels). The task difficulty in deceleration compared to acceleration is associated with this hysteresis effect in the precuneus. In this case also, the rate of tapping in deceleration > rate of tapping in acceleration, showed brain activations in the contra- and ipsilateral posterior cerebellum, the left superior temporal gyrus and the right parahippocampal gyrus (Fig. 6). Figure 7 shows brain activations during the continuous case associated with SMS in the acceleration phase versus deceleration phase. Figure 8 shows the activations for SMS during continuous versus discrete cases of the acceleration phase.

## Discussion

This study investigated the brain's inertia for movement during a SMS task in an fMRI. The behavioral responses and brain activations were different for accelerating and decelerating phases of the SMS task. This paradigm involved perception of machine-cued rhythms and responsive motor coordination. Thus these results contribute to our understanding of the brain mechanisms involved in some aspects of perceptual decision-making [46], motor coordination [47], and our perceptual and behavioral performance limits. The SMS task used here recruited the brain regions commonly affected in movement disorders, such as the substantia nigra. These findings thus may have a therapeutic value to those suffering from a variety of neurological disorders. Based on the current trend in fMRI research, the number of subjects included in the study could be towards the lower end. However, we expect these findings to hold true at a higher significance level for a larger pool of subjects, as individual performance analysis showed each subject performed better in the acceleration than deceleration cases.

### Task difficulty and behavioral performance

Synchrony indices (Fig. 2 and Fig. 3) allowed insight into the difficulty of each variation of the task. A greater sync index denotes a higher accuracy in the task, and therefore provides a basis for evaluating how challenging each phase was for the subjects. In the continuous case, each subject performed better during the accelerating phase than the decelerating phase. This is in agreement with the participants' self-evaluation immediately after completing the study. Subjects stated that following a decelerating rhythm was much more challenging. Focusing only on the accelerating phases, all subjects had a greater synchrony index in the continuous case than the discrete case. Further, during continuous acceleration phases subjects had a significantly greater synchrony index than during discrete acceleration phases. In discrete cases, there was no significant difference between accelerating and decelerating average synchrony indices. The subjects' perception of cued rhythms and behavioral responses give insight into why the brain's state was different for acceleration and deceleration phases. Considering this SMS task as a simple stimulus- response sequence, during the acceleration phase, the stimulus always arrived slightly earlier than expected, and thus triggered a response immediately. During the deceleration phase however, each stimulus was delayed and the subject had to actively inhibit their tendency to respond until the stimulus arrived. The factor that contributed most to the discrepancy in the behavioral performance between the acceleration and deceleration phases could be the necessary controlled delay in response for the decelerating phases. The associated brain responses can be expected to depend on the direction (acceleration or deceleration) of the rhythm rate variation, suggesting a history-dependent activity level – the hysteresis effect. These findings support a previous study [23] and are consistent with history-dependent perceptual effects [30]. The two cases, discrete and continuous variations of rhythm rate, can represent discrete and continuous movements as previously defined, see for example, [7], [33]. Discrete movements are believed to require more brain resources for timing control mechanisms than continuous movements [33]. Consistent with these studies, our experiment showed that subjects experienced more difficulty in SMS with metronomes during discrete rhythm changes than continuous rhythm changes.

### Brain activations

#### Task versus rest activity

In the task (continuous and discrete cases) versus rest contrast the greatest activity was shown in the left precentral gyrus and the right substantia nigra (STN) (Fig. 4). As we know from previous studies on finger movements, simple to complex types of finger tapping can activate various cortical and subcortical structures including the primary motor, premotor, parietal areas, thalamus, cerebellum, and the basal ganglia [48]–[57]. Each structure or group of structures plays a specific role in the planning, initiating, executing, timing and sequencing of movements. The premotor and primary motor areas are known for planning and executing movements [13]. The basal ganglia, cerebellum, and thalamus are considered important for sequencing and timing of movements [6], [58]. The STN, a structure within the basal ganglia circuitries, is known to be important for voluntary movement [55], perception of timing [12], [59], the initiation of motor sequences [55], and is a clinically important site for the dysfunction of dopamine release characteristic of Parkinson's disease (PD). Results of our SMS task reveal STN activity to be associated with rhythm perception, initiation and actual execution of movements. The STN activity for this SMS task lends potential use in diagnosis or therapy for PD patients.

#### Deceleration versus acceleration activity

In the deceleration versus acceleration contrast from the continuous case of the task, significant activity is seen in the left precuneus (Fig. 5 (A)). Precuneus activity has been associated with visuo-spatial and memory-related cognitive activities [19] and motor coordination [60]. During motor tasks, the precuneus was found to be active during sequencing experiments with its activity being modulated by sequence complexity and length. During bimanual motor coordination, the precuneus region immediately posterior to the cingulate gyrus was activated [60]. This section of the precuneus has previously been reported as active during the execution or imagination of spatially demanding tasks [19]. In patients with PD, precuneus activity is increased compared to healthy controls during bimanual movements [61]. Anatomically, the precuneus has widespread connectivity, involving higher association cortical and subcortical structures [19]. Taken together, the precuneus activity for synchronizing to visual cues in our study could be associated with the additional motor coordination effort that requires highly integrated and associative information.

Further examining the precuneus activity for deceleration versus acceleration phases reveals the hysteresis effect. Figure 5 (A) shows the percent BOLD changes during acceleration and deceleration phases according to the rate of tapping. As shown in Fig. 5 (B), under 1 Hz, there is no significant difference in percent BOLD signal change, however, above 1 Hz and overall, the deceleration shows significantly greater percent change. These results suggest that the brain has a 'cognitive' inertia that depends on the direction of the behavioral or sensory input manipulation. From these findings of BOLD response alone, we are not able to definitively pinpoint whether the nonlinearity in brain BOLD response is due to neuronal activity, hemodynamic activity, or both. However, based on the observed brain – behavior relation (as in synchrony index or tapping rate and brain activity) in this experiment, the nonlinearity seems to have a definite neural origin. The overall effect of nonlinearity however is likely due to both neuronal and hemodynamic sources. The additive nature of hemodynamic response at the higher (>2 Hz) tapping rates we had could also contribute. Recent fMRI study has also shown that a distributed network of brain areas is involved in hysteresis [62]. Perceptual hysteresis was linked to fMRI BOLD responses in certain brain regions during visual letter recognition [30]. These results suggest activity in the precuneus is partially dependent on the history or previous activation (whether remnant from a higher tapping rate- deceleration, or lower tapping rate-acceleration). Precuneus activity hysteresis is consistent with the previously reported results [23], and is further supported behaviorally by our subjects' self-reports that slowing down was more difficult than speeding up their tapping in synchrony to the metronome. The hysteresis effect, as observed in the present study and others [30], can be shown to occur in simulations of BOLD responses if the effect is present at the neural level (the simulation results are not shown here). In the context of our data, hysteresis refers to the fact that over the range of identical tapping frequencies, the percent BOLD signal change in the precuneus was different when the subject was completing an accelerating phase versus a decelerating phase. The physical parameters of the visual inputs remained exactly identical for acceleration and deceleration, but the perceived difficulty differed which is a strong signature of hysteresis and possible evidence of the brain's inertia for movements.

#### Tapping rate-related activity

A contrast between the decelerating versus the accelerating rates of the continuous case was done to isolate structures involved in solely the more difficult task of SMS with a decelerating rhythm. The contrast reveals right and left cerebellar vermis, superior temporal gyrus, and parahippocampal gyrus activations (Fig. 6). The cerebellar activity remains consistent with previous studies identifying these areas to be involved in finger tapping [54], [63]. Extra activation during the decelerating task may signify an enhanced role of the cerebellum in externally paced rhythmic finger movements [58], [64]. The cerebellum has been shown to engage in temporal control of repetitive movements, sensory perception, and coordination [63]. SMS tasks have activated the superior temporal gyrus in previous studies [65], [66]. The parahippocampal cortex has been tied to processing contextual associations [67]–[69], which might explain its activation in processing a rhythm or pattern. Prior studies have related parahippocampal activity with performance on working memory tasks [70]. The differences in activity support extant data that greater activity in the motor system correlates with difficulty and complexity of the performed sequence [71]. Slowing down finger tapping was more challenging for subjects as more areas of the brain were recruited to complete the task and behavioral performance accuracy was significantly less than in acceleration phases.

Lutz and colleagues found that subjects often internally generate their movements before actually perceiving the visual stimulus, in an effort to maximize synchronization. This pacing is disrupted with irregular tapping, and thus reaction times are more broad [72]. Thus it can be expected that finger tapping with an irregular rhythm (such as accelerating or decelerating rates) will produce a range of reaction times and require stronger participation of motor areas. This study goes a step further, finding that there are differences even within the irregular paced rhythms (whether accelerating or decelerating and whether sinusoidal or stepwise). Previous study has proposed that different mechanisms may be at work for processing slow versus fast movements [73]. In this study, Jancke and colleagues found that slow movements demanded extensive neural activity to continuously compare motor commands with afferent information. In contrast, fast finger movements were found to be controlled by a program-like mode that predicted motor commands prior to afferent feedback [73]. These theories support the findings that SMS with accelerating rhythms recruit less brain regions than decelerating rhythms. Our study adds to these findings by uniquely observing these mechanisms at work during a sinusoidal and stepwise increase and decrease of tapping rhythms.

#### Synchronizing performance-related activity

This contrast was conducted to illustrate the areas of the brain associated with the SMS tasks, and further show the additional activations associated with the more challenging decelerating phases and discrete phases. The synchronization index score was significantly higher for acceleration; therefore, these results isolate the regions associated with the more difficult deceleration phase. Brain activation for synchrony index in acceleration versus synchrony index in deceleration during continuous cases includes the medial frontal gyrus, uncus, occipital lingual gyrus, and the parahippocampal gyrus (Fig. 7). These areas of the brain have been closely associated with SMS. Every subject's average synchrony indices were greater for the acceleration phases, meaning that their synchronization-accuracy was higher than it was during decelerating phases. Activation in the occipital lingual gyrus has been associated with visuospatial/visuomotor memory [74], while activity in the uncus, part of the parahippocampal gyrus could be related to contextual working memory. The medial frontal gyrus has been specifically associated with instruction-related activity rather than the motor-execution related activity [75]. This region is likely reflective of the analysis of sensory signals and decisions on motor commands [75].

Contrasting specifically the accelerating phases of the continuous case with the accelerating phases of the discrete (step-like) case (synchrony index continuous versus synchrony index discrete) revealed activity in the middle frontal gyrus, medial frontal gyrus, precuneus, and posterior cingulate cortex (Fig. 8). The precuneus and frontal gyrus have been discussed above, as they were visible in other contrasts as well. The precuneus has been widely linked to visuospatial memory [76]. Using synchrony indices as a measure of accuracy and thus difficulty, it can be inferred that the discrete acceleration phase was more challenging than the continuous acceleration phase.

Aschersleben described a tendency of subjects to reestablish the negative asynchrony between the cue and finger tap, which arises due to differences in peripheral and/or central processing times [39]. This intrinsic tendency supports our findings that SMS with accelerating rhythms is easier than with decelerating rhythms. Slowing down tapping speed opposes the described predictive tendency, thus we found poorer synchronization accuracy for decelerating rhythms. While a widely cited phenomenon, the underlying mechanisms of this error are still not completely understood. This study hopes to shed light on the structures involved, so that the physiological process may be better understood.

## Conclusions

This study suggests that the brain's inertia for movement is different for acceleration and deceleration. During a SMS task, participants' behavioral performance was consistently dissimilar for accelerating and decelerating phases of motor movements. The brain activity was greater in the precuneus during deceleration than during acceleration when rates were changed following a smooth (continuous) sinusoidal curve from 0.5 to 3 Hz. The precuneus activity showed a significant hysteresis effect for rates above 1 Hz. Here, the hysteresis effect is a quantifiable measure of the brain's inertia difference between deceleration and acceleration. This result of the precuneus activity is consistent with its role in a visual perception and motor action that requires highly integrated and associative information [19], [60], [61]. A network of distributed brain activity associated with the movement rate and synchrony index further supported the brain's inertia difference between acceleration and deceleration phases of the movement task. The tapping rate during deceleration compared to acceleration recruited activity in the cerebellum, superior temporal gyrus, and parahippocampal gyrus. The synchrony of movements with the metronome recruited the occipital, prefrontal and hippocampal regions during the continuous acceleration phase compared to continuous deceleration. The occipital, prefrontal, and precuneus regions were recruited during continuous acceleration compared to discretely changing acceleration. These results altogether provide evidence towards a relationship between the brain's inertia and perceptual or behavioral performance limits. This study contributes to our better understanding of neural mechanisms for various aspects of sensorimotor synchronization, perception-action including rhythm perception, perceptual decision-making and motor coordination.
